# Effect on attendance by including focused information on spirometry in preventive health checks: study protocol for a randomized controlled trial

**DOI:** 10.1186/s13063-016-1704-7

**Published:** 2016-12-01

**Authors:** Lene Maria Ørts, Anders Løkke, Anne-Louise Bjerregaard, Helle Terkildsen Maindal, Annelli Sandbæk

**Affiliations:** 1 Department of Public Health, Section for General Practice, Aarhus University, Bartholins Allé 2, 8000 Aarhus, Denmark; 2 Department of Respiratory Diseases, Aarhus University Hospital, Nørrebrogade 44, 8000 Aarhus C, Denmark; 3 Department of Public Health, Section of Health Promotion and Health Services, Aarhus University, Bartholins Allé 2, 8000 Aarhus, Denmark

**Keywords:** Spirometry, Preventive health check, Attendance

## Abstract

**Background:**

Early detection of lung diseases can help to reduce their severity. Lung diseases are among the most frequently occurring and serious diseases worldwide; nonetheless, many patients remain undiagnosed. Preventive health checks including spirometry can detect lung diseases at early stages; however, recruitment for health checks remains a challenge, and little is known about what motivates the attendance.

The aim of the study is to examine whether focused information on spirometry in the invitation compared to general information will impact the attendance rate in preventive health checks.

**Methods/design:**

This randomized, controlled trial tests the effect of information on spirometry embedded in the Check your Health Preventive Program (CHPP). The CHPP is an open-label, household cluster-randomized, controlled trial offering a preventive health check to 30- to –49-year-olds in a Danish municipality from 2012 to 2017 (*n* = 26,216). During 2015–2016, 4356 citizens aged 30–49 years will be randomized into two groups. The intervention group receives an invitation which highlights the value and contents of spirometry as part of a health check and information about lung diseases. The comparison group receives a standard invitation containing practical information and specifies the contents of the general health check.

Outcomes are (1) differences in attendance rates measured by the proportion of citizens attending each of the two study groups and (2) proportion of persons at risk defined by smoking status and self-reported lung symptoms in the study groups. The proportion of participants with abnormal spirometry assessed at the preventive health check will be compared between the two study groups.

**Discussion:**

The results from the present study will inform future recruitment strategies to health checks. The developed material on content, value, and information about lung disease is feasible and transferable to other populations, making it easy to implement if effective.

**Trial registration:**

ClinicalTrials.gov: NCT02615769. Registered on 25 November 2015.

**Electronic supplementary material:**

The online version of this article (doi:10.1186/s13063-016-1704-7) contains supplementary material, which is available to authorized users.

## Background

Early detection of lung diseases can help reduce the severity of these diseases when inflicted. Lung diseases are among the most frequently occurring and serious conditions in the western world, including Denmark [1]. The Danish Health Authority recommends spirometry every second year to all individuals older than 35 years with at least one respiratory symptom and/or exposure to any known risk factor (smoke, genetics, air pollution, chemical fumes, or dust) in order to facilitate early case finding of chronic obstructive pulmonary disease (COPD) [2]. However, an estimated 200,000 Danish citizens (50%) with COPD remain undiagnosed and unknown to the health care system [2, 3].

Preventive health checks including spirometry can be used to detect lung diseases at early stages. However, a well-known challenge is low uptake, especially in people at risk of developing disease [4]. Previous studies of attendees in preventive health checks [5] reported average attendance rates from 18 to 82%. They also showed that non-attendees were less healthy than the attendees and that those least likely to attend health checks were younger, single men with low income or low socioeconomic status, active smokers, or the unemployed or less educated [5]. Little is known about what motivates the attendance to health checks.

In a systematic review by Jepson et al., interventions used to increase uptake in screening for a variety of conditions are compared in 190 trials [6]. These interventions targeted individuals and revealed enhanced invitation procedures. An appointment in the invitation letter, telephone calls, and reminders seemed to increase the participation rate effectively [6]. A few studies evaluated the effect of providing additional information in the invitation. Kiernan et al. examined three strategies of recruiting citizens into trials by personalizing the invitation letter and by adding additional information. They found that a personalized letter including heart disease risk statistics did not yield a higher response rate than the standard letter [7]. Sallis et al. compared an enhanced invitation letter applying behavioral insights with a standard invitation letter and found a difference in the participation rate from 29.3% to 33.5% [8]. Van Wonderen et al. found that neither the rank of the individual signing a subsequent detailed information letter nor the use of an official funding agency logo on the study’s initial invitation made an impact on patient attendance [9]. Finally Martinson et al. showed a difference in response rate from 55% to 69% using monetary incentives for recruiting adolescents to a trial focusing on smoking cessation [10].

In studies aiming to improve participation in colorectal cancer screening, enhanced invitation letters and leaflets have yielded an increase in participation of up to 6% [11, 12]. Recently, Quaife et al. [13] published a study protocol to test the hypothesis that targeted invitation strategy will increase the uptake in a lung cancer screening compared with a standard invitation. This population consisted of patients aged 60–75 with a known smoking history. To our best knowledge, no trials aimed to increase the attendance in general health checks by including information on the benefits of measuring spirometry, and only a few studies have included spirometry in a preventive health check [14, 15].

The aim of the present study is to examine whether focused information about spirometry in the invitation material will influence the attendance rate in preventive health checks. We also aim to describe the characteristics of the attendees and non-attendees. We hypothesize that information on spirometry as part of the invitation material for the preventive health check will increase the overall attendance rate by 5 percentage points and also that more people at risk will attend. The study protocol conforms to the Consolidated Standards Of Reporting Trials (CONSORT) statement [16] and the Standard Protocol Items: Recommendations for Interventional Trials (SPIRIT) 2013 statement [17]. See Additional files 1 and 2 [17]. This paper will follow the CONSORT extension for non-pharmacological interventions and cluster trials [18, 19].

## Methods/design

### Trial design

The trial is an open-label, household cluster-randomized, controlled trial with a two-group parallel design. The trial is embedded in the fourth year (2015–2016) of the ongoing Check your Health Preventive Program (CHPP) [20]. The CHPP offers a preventive health check to all 30- to 49-year-old citizens in a Danish municipality during the years 2012–2017 (*n* = 26,216).

We began designing the present study in January 2015, the recruitment started in November 2015, and we expect it to be completed in December 2016.

### Population

**Fig. 1:**
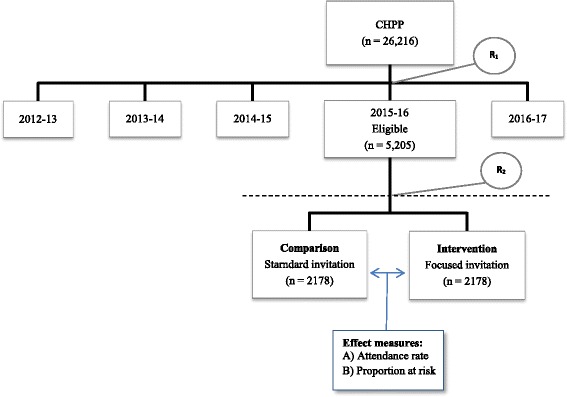
Flowchart of participants in the Check your Health Preventive Program [20] and the present trial. *R*_*1*_ household randomization into five groups of equal size, *R*_*2*_ household randomization into the intervention group or comparison group in the present study before sending of the invitations

The citizens are randomized by household into five groups of equal size, each representing the specific year they are invited to attend a health check. Citizens randomized for group 4 (*n* = 5205) are eligible for the present trial (Fig. 1). The Danish Civil Registration System (CRS) [21, 22] was used to identify all citizens living in the municipality of Randers aged 30–49 years on 1 January 2012 included in the CHPP. The CRS is an administrative register containing individual-level information including the unique and permanent ten-digit civil personal registration (CPR) number on all persons residing in Denmark [21, 22]. The only exclusion criterion is terminal illness as reported by a general practitioner [20].

### Setting

The health checks and the subsequent health behavioral programs are conducted at a local health care center. The subsequent health interview takes place at the participant’s own general practitioner’s office. To ensure standardization and quality, the health checks are conducted by health professionals experienced in risk communication and conduction measurement procedures.

### Questionnaire

Prior to the health check, the participants are asked to answer a web-based questionnaire regarding self-rated health [23], mental health [23], alcohol consumption [24], physical activity, and smoking habits using items from the Danish National Health Profile questionnaire [25]. The question concerning lung symptoms is a modified version of the question from the Clinical COPD Questionnaire [26] (Table 1). The participants are also asked which element of the preventive health check has their highest interest (not shown).

**Table 1:** Items and response categories from the questionnaire

Do you smoke?	• Yes, daily • Yes, at least once a week • Yes, occasionally (part-time smoker) • No, I quit in (year) • No, I have never smoked
On average, how much do you smoke daily?	• Number of cigarettes • Number of cheroots • Number of cigars • Number of pipes
During the past 4 weeks, how much of the time were you troubled by dyspnea, wheezing or coughing? (only one x)	• All of the time • Most of the time • Now and then • Rarely • Not at all

### Spirometry

Lung function will be assessed using the EasyOne Diagnostic Spirometer (Ndd Medical Technologies, Andover, MA, USA), which is calibrated daily. The spirometer measures forced expiratory volume in one second (FEV_1_) and forced vital capacity (FVC). Spirometer software calculates the FEV_1_/FVC ratio and the predicted value of FEV_1_ based on reference values. At each examination, the criterion for correct procedure performance is at least three measurements differing by less than 5%. Reversibility using a bronchodilator is not allowed. Abnormal spirometry is defined as FEV_1_/FVC <0.7 or FEV_1_% pred. <0.8 or FVC % pred. <0.8. The spirometric cut-off values used in the Global Initiative for Chronic Obstructive Lung Disease (GOLD) guidelines [27, 28] will be used for classifying disease severity.

### The health check

In addition to spirometry, the clinical examination includes the following measurements: blood analysis of total cholesterol, low-density lipoprotein (LDL), glycated hemoglobin (HbA1c), systolic and diastolic blood pressure, bodyweight, height, and Astrand’s submaximal bike test (cardiorespiratory fitness) [20, 29]. After the health check, the results are presented in a profile pamphlet including recommendations for follow-up according to the citizen’s risk profile. Citizens with abnormal spirometry are recommended to consult their general practitioner for further examination. Execution of the health check is standardized by a written standardized operational procedure (SOP), and adherence is monitored continuously. The average attendance rate in the CHPP was 53% during 2012–2015.

### National registers

In Denmark we have a unique opportunity to link study measurements to nationwide medical registers and databases [21, 22]. To understand the processes involved in responding to the intervention, especially among the non-attendees, secondary characteristics regarding sociodemographics, redeemed medication, and consultations at their general practitioner will be obtained by linking to Danish registers [21, 30, 31].

### The routine procedure for invitation

The comparison group (*n* = 2178) receives a standard invitation and a leaflet, as shown in Additional file 3. The leaflet and invitation contain practical information and specify the contents of the general health check [20]. The invitation also refers to the homepage for further information. Letters are dispatched continuously with a prefixed appointment for the health check. The appointment can be accepted, modified, or rejected via the Internet or by phone. A reminder is sent out with a new appointment time, if the appointment is not accepted within 7 days. Failure to reply within 3 weeks procures another reminder.

### The intervention

The intervention group (*n* = 2178) receives a modified invitation and a leaflet which highlights the benefits of spirometry as part of a health check and provides information about how to prevent lung diseases. The invitation was developed in collaboration with the Danish Lung Association and was tested for content and comprehensibility on a focus group before dispatch of the invitations to the intervention group. The citizens are recommended to visit the homepage for further information and advice on how to prevent lung diseases [32]. All the material can be accessed in Additional file 3.

### Randomization

Randomization is performed on clusters defined by households based on addresses from the Danish Civil Register [21, 22]. Furthermore, the randomization is balanced at the general practitioner (GP) level to ensure distribution of the follow-up workload over 5 years. Using the same index individual per households, 4356 citizens randomized to group 4 in the CHPP are further randomized by household into two parallel groups: intervention or comparison (Fig. 1). Randomization is handled by an independent statistician. Implementation and booking are handled by a data manager. The intervention, the outcome, and the group assignment are not revealed to the participants, And the health care professionals also remain blinded. The blinding will be unlocked as the last spirometry measurement is done in December 2016.

### Outcome

The outcomes are (1) differences in attendance rates measured by the proportion of citizens attending each of the two study groups and (2) proportion of persons at risk defined by smoking status and self-reported lung symptoms in the study groups. Furthermore, the characteristics of the two groups will be described according to sex, age, smoking history, and self-reported lung symptoms. Smoking status will be classified as smoker, ex-smoker, or never a smoker. The proportion of participants with abnormal spirometry assessed at the preventive health check will be compared between the two study groups.

### Sample size

The sample size is estimated at 4356 participants in total and is calculated on the basis of the following assumptions: 1:1 randomization, two-sided significance level of 0.05, power of 0.9, intra-cluster correlation coefficient of 0.01; and with an average of 1.5 citizens in each household, we calculated the design effect [33] to be 1.05. Calculations are further based on the objective of being able to detect a difference of 5 percentage points in effect size based on an attendance rate of 53–58%. The investigators determined this 5 percentage point difference to be clinically meaningful based on expert opinion [7–9, 34] and based on the run-in period where the participation rate was 53%. Also, an increase of 5 percentage points would deliver clinically meaningful benefit if scaled in a nationwide program. In order to reflect a possible clustering effect of the general practitioners (*n* = 46), which seldom exceeds 0.01 in primary care settings [35], the intra-cluster correlation coefficient was included. To limit the missing data in our study [36], incomplete questionnaires and incomplete spirometry measurements will be excluded. Non-attendees will be described with information supplied from national registers [21, 22]. A sensitivity analysis will also be performed.

### Statistics

The effect of the intervention on attendance will be estimated by the difference of the proportion of citizens attending each of the groups. Furthermore, a descriptive analysis of attendees and non-attendees will be performed regarding sex, age, smoking history, self-reported lung symptoms, and spirometry measurements. The analysis will follow the intention-to-treat principle [16].

For the descriptive part of this study, bivariate and multivariate analyses will be performed. Numerical variables are presented as mean ± standard deviation, and binary variables as absolute numbers and relative (percent) frequencies. Student’s *t* test will be used when comparing means or changes in means of numerical variables, and Fisher’s exact *t* test or the chi-squared test will be used when comparing proportions for binary variables. Binary outcomes will be analyzed with logistic regression. The statistical significance level is set at *p* < 0.05. Statistical analysis will be performed using Stata 14.0 software [37]. A detailed Statistical Analysis Plan will be finalized before access to the data and will be attached to the ClinicalTrials.gov trial identifier (NCT02615769).

## Discussion

This study is the first to examine the benefits of a low-cost and enhanced invitation strategy to identify lung impairment among young adults in a real-world setting. It is designed as a pragmatic and randomized study and will provide information which can be used directly by health care planners in the decision of how to implement health checks. The outcomes were chosen to mirror the aims of the intervention: the overall attendance rate and the attendance of people at risk for lung impairment.Our recruitment strategy is to reach the citizens in three different ways: by webpage, leaflet, and invitation letter in a randomized controlled design. Prior studies have shown an increase in attendance by enhancing invitation material [9, 11, 12, 38]. Therefore, we expect an increase in the attendance of 5 percentage points, and we also expect that more people at risk will attend. We believe it is a realistic goal, even though previous studies indicate that smokers feel ashamed and guilty of their self-inflicted disease and therefore hesitate to seek help [4]. In order to avoid having smokers absent themselves, when we were designing the new material for the intervention group, we involved a communications consultant from the Danish Lung Association [39], who is an expert in content and comprehensibility.

### Strengths and limitations

Participants will be enrolled directly from the general population; therefore, they will not receive any screening prior to inclusion. Thus, the generalizability will not only reflect certain at-risk populations such as current smokers, prior smokers, or citizens with chronic disease. The high transferability of the invitation material and the application of the intention-to-treat principle provide a high external validity. Moreover, the objective measure of attendance contributes with a high internal validity. We chose cluster randomization by household to limit the expected contamination between attendees living together, because motivation to attend a health check will potentially impact the entire household. To avoid having attendees refuse to participate, households are invited together and two reminders are sent out. Furthermore, appointments are scheduled outside of work hours. Finally, we have the unique opportunity to use the Danish registers as a population-based health care database with information about the non-attendees [21].

The present study has some limitations. Firstly, we are aware that both groups have the same accessibility to the webpage regarding how to prevent lung disease, and this may dilute the difference between the two groups. Secondly, the right level of extra information is difficult to achieve. Too much information may cause the participants to believe that the study is only about lung function and possibly scare some of the citizens at risk. On the other hand, too little preventive information may dilute the intervention. Thirdly, we have no spirometry measurements on the non-attendees, which complicates the comparison among the non-attendees and the attendees. Nonetheless, we have the opportunity to supply the characteristics of the non-attendees by redeemed medication and sociodemographic characteristics by the Danish registers [21, 31]. Although our population is fairly young, the most severely affected and chronic patients will not show up for the examination as it requires some physical and mental effort, but we expect the effect to be equal among the two groups. Finally, there is a risk of misclassification among, e.g., citizens with flu or pneumonia, thus resulting in false positive outcomes. However, this risk is expected to be equal among the two groups due to the randomization.

### Perspectives

Lung diseases represent a significant burden for patients and health care systems worldwide. Optimizing early detection followed by pharmacological and non-pharmacological interventions can not only improve a patient’s health status and quality of life, but can also reduce health care expenses. The results from the present study are expected to contribute important knowledge about the value of information on spirometry in invitations to health checks.

### Trial status

At the time of submission of this manuscript, the trial has enrolled approximately 3500 participants. Recruitment is ongoing.

## Additional files

Additional file 1: SPIRIT 2013 Checklist: recommended items to address in a clinical trial protocol and related documents*. (DOC 121 kb)
Additional file 2: SPIRIT figure. (DOC 48 kb)
Additional file 3: Invitation, leaflet, and website text to the invitation and comparison group. (PDF 4057 kb)

## Abbreviations

CHPP: Check your Health Preventive Program
CPR: Civil personal registration
CRS: Danish Civil Registration System
FEV_1_: Forced expiratory volume in one second
FVC: Forced vital capacity
GP: General practitioner
RCT: Randomized controlled trial

## References

[1] Registry of Causes of Death. Available at: http://sundhedsdatastyrelsen.dk/dar. Accessed 14 Oct 2015.

[2] Chronic obstructive pulmonary disease. Recommendations for early identification, follow-up, treatment and rehabilitation. Copenhagen, Denmark: The National Board of Health; 2007.

[3] Løkke A, Ulrik CS, Dahl R (2012). Detection of previously undiagnosed cases of COPD in a high-risk population identified in general practice. COPD.

[4] Halding A-G, Heggdal K, Wahl A (2011). Experiences of self-blame and stigmatisation for self-infliction among individuals living with COPD. Scand J Caring Sci.

[5] Dryden R, Williams B, McCowan C, Themessl-Huber M (2012). What do we know about who does and does not attend general health checks? Findings from a narrative scoping review. BMC Public Health.

[6] Jepson R, Clegg A, Forbes C, Lewis R, Sowden A, Kleijnen J (2000). The determinants of screening uptake and interventions for increasing uptake: a systematic review. Health Technol Assess.

[7] Kiernan M, Phillips K, Fair JM, King AC (2000). Using direct mail to recruit Hispanic adults into a dietary intervention: an experimental study. Ann Behav Med.

[8] Sallis A, Bunten A, Bonus A, James A, Chadborn T, Berry D (2016). The effectiveness of an enhanced invitation letter on uptake of National Health Service Health Checks in primary care: a pragmatic quasi-randomised controlled trial. BMC Fam Pract.

[9] van Wonderen KE, Mohrs J, IJff M, Bindels PJE, ter Riet G (2008). Two simple strategies (adding a logo or a senior faculty’s signature) failed to improve patient participation rates in a cohort study: randomized trial. J Clin Epidemiol.

[10] Martinson BC, Lazovich D, Lando HA, Perry CL, McGovern PG, Boyle RG (2000). Effectiveness of monetary incentives for recruiting adolescents to an intervention trial to reduce smoking. Prev Med (Baltim).

[11] Hewitson P, Ward AM, Heneghan C, Halloran SP, Mant D (2011). Primary care endorsement letter and a patient leaflet to improve participation in colorectal cancer screening: results of a factorial randomised trial. Br J Cancer.

[12] Wardle J, Williamson S, McCaffery K (2003). Increasing attendance at colorectal cancer screening: testing the efficacy of a mailed, psychoeducational intervention in a community sample of older adults. Health Psychol.

[13] Quaife SL, Ruparel M, Beeken RJ (2016). The Lung Screen Uptake Trial (LSUT): protocol for a randomised controlled demonstration lung cancer screening pilot testing a targeted invitation strategy for high risk and “hard-to-reach” patients. BMC Cancer.

[14] Lauritzen T, Leboeuf-Yde C, Lunde IM, Nielsen KD (1995). Ebeltoft project: baseline data from a five-year randomized, controlled, prospective health promotion study in a Danish population. Br J Gen Pract.

[15] Lange P, Celli B, Agustí A (2015). Lung-function trajectories leading to chronic obstructive pulmonary disease. N Engl J Med.

[16] Moher D, Hopewell S, Schulz KF (2010). CONSORT 2010 Explanation and Elaboration: updated guidelines for reporting parallel group randomised trials. J Clin Epidemiol.

[17] Chan A-W, Tetzlaff JM, Gøtzsche PC (2013). SPIRIT 2013 explanation and elaboration: guidance for protocols of clinical trials. BMJ.

[18] Boutron I, Moher D, Altman DG, Schulz KF, Ravaud P (2008). Extending the CONSORT statement to randomized trials of nonpharmacologic treatment: explanation and elaboration. Ann Intern Med.

[19] Campbell MK, Piaggio G, Elbourne DR, Altman DG (2012). Consort 2010 statement: extension to cluster randomised trials. BMJ.

[20] Maindal HT, Støvring H, Sandbaek A (2014). Effectiveness of the population-based Check your health preventive programme conducted in primary care with 4 years follow-up [the CORE trial]: study protocol for a randomised controlled trial. Trials.

[21] Schmidt M, Pedersen L, Sørensen HT (2014). The Danish Civil Registration System as a tool in epidemiology. Eur J Epidemiol.

[22] Pedersen CB (2011). The Danish Civil Registration System. Scand J Public Health.

[23] Ware J, Kosinski M, Keller SD (1996). A 12-Item Short-Form Health Survey: construction of scales and preliminary tests of reliability and validity. Med Care.

[24] Saunders JB, Aasland OG, Babor TF, de la Fuente JR, Grant M (1993). Development of the Alcohol Use Disorders Identification Test (AUDIT): WHO Collaborative Project on Early Detection of Persons with Harmful Alcohol Consumption--II. Addiction.

[25] Christensen AI (2013). Den nationale sundhedsprofil 2010: Hvordan har du det?.

[26] van der Molen T, Willemse BWM, Schokker S, ten Hacken NHT, Postma DS, Juniper EF (2003). Development, validity and responsiveness of the Clinical COPD Questionnaire. Health Qual Life Outcomes.

[27] Vestbo J, Hurd SS, Agusti AG (2013). Global strategy for the diagnosis, management, and prevention of chronic obstructive pulmonary disease: GOLD executive summary. Am J Respir Crit Care Med.

[28] Vestbo J, Hurd SS, Rodriguez-Roisin R (2012). The 2011 revision of the global strategy for the diagnosis, management and prevention of COPD (GOLD)—why and what?. Clin Respir J.

[29] Astrand I (1960). Aerobic work capacity in men and women with special reference to age. Acta Physiol Scand Suppl.

[30] Statistican Denmark. No Title. (February 2016). http://www.dst.dk/da/. Accessed 13 Oct 2015

[31] Kildemoes HW, Sørensen HT, Hallas J (2011). The Danish National Prescription Registry. Scand J Public Health.

[32] Check your health preventive program. https://sundhedscenter.randers.dk/tjek-dit-helbred/. Accessed 20 May 2016.

[33] Donner A, Klar N (2000). Design and analysis of cluster randomization trials in health research.

[34] Mapstone J, Elbourne D, Roberts IG (2007). Strategies to improve recruitment to research studies. Cochrane Database Syst Rev.

[35] Adams G, Gulliford MC, Ukoumunne OC, Eldridge S, Chinn S, Campbell MJ (2004). Patterns of intra-cluster correlation from primary care research to inform study design and analysis. J Clin Epidemiol.

[36] Little RJ, Agostino RD, et al. The prevention and treatment of missing data in clinical trials. N Engl J Med. 2012;367(14):1355–60. doi:10.1056/NEJMsr1203730. 10.1056/NEJMsr1203730 PMC3771340 23034025

[37] Stata: Data Analysis and Statistical Software. http://www.stata.com/. Accessed 24 Oct 2015.

[38] Kølner-augustson L, Thøgersen N, Faaborg TH, Weinreich UM (2015). The majority of participants with abnormal spirometry at walk-in consult their general practitioner as recommended. Dan Med J.

[39] The Danish Lung Association. (Feb 2016). https://www.lunge.dk/about-us. Accessed 15 Feb 2016.

