# Supplementary material for: Effect on attendance by including focused information on spirometry in preventive health checks: study protocol for a randomized controlled trial
Source: Trials. 2016 Dec 1;17:571. doi: 10.1186/s13063-016-1704-7 (PMC5134092; doi:10.1186/s13063-016-1704-7)
Supplement: Additional file 2: — SPIRIT figure. (DOC 48 kb) [file 13063_2016_1704_MOESM2_ESM.doc]

SPIRIT Figure

|  | **STUDY PERIOD** | | | |  |
| --- | --- | --- | --- | --- | --- |
|  | **Enrolment** | **Allocation** | **Received invitation** | **Visit at health care center** | **Visit at GP* if needed** |
| **TIMEPOINT** | ***-2 weeks*** | **0** | ***2 weeks*** | ***4 weeks*** | ***6 weeks*** |
| **ENROLMENT:** |  |  |  |  |  |
| **Eligibility screen** | X |  |  |  |  |
| **Informed consent** |  |  | X | X |  |
| ***Randomization*** | X |  |  |  |  |
| **Allocation** |  | X |  |  |  |
| **INTERVENTIONS:** |  |  |  |  |  |
| ***Intervention – invitations sent out*** |  |  | X |  |  |
| ***Intervention –***  ***Leaflet sent out*** |  |  | X |  |  |
| **ASSESSMENTS:** |  |  |  |  |  |
| ***Questionnaire*** |  |  | X | X |  |
| ***Clinical measurements*** |  |  |  | X | X |
| **Register data** | X |  | X | X |  |
| ***Attendance rate*** |  |  |  | X |  |

*GP: general practitioner.
