# Supplementary material for: Effect on attendance by including focused information on spirometry in preventive health checks: study protocol for a randomized controlled trial
Source: Trials. 2016 Dec 1;17:571. doi: 10.1186/s13063-016-1704-7 (PMC5134092; doi:10.1186/s13063-016-1704-7)
Supplement: Additional file 3: — Invitation, leaflet, and website text to the invitation and comparison group. (PDF 4057 kb) [file 13063_2016_1704_MOESM3_ESM.pdf]

Peter Petersen
Vestergade 5
8900 Randers C

Randers, 1st of January 2016

Free health check (Headline for the comparison group)

Get a free health and lung check (Headline for the intervention group)

(The remaining document is the same for both groups)

You are invited for a health examination at Randers Health Centre and a consultation with your general practitioner if necessary.

We have made an appointment for you (remember to confirm or make a new appointment)

Remember to confirm your appointment

Friday the 1st of January 2016 at 10 am
Randers Health Centre
Biografgade 3, 3rd floor, 8900 Randers C

The appointment must be confirmed or changed **no later than** 1st of January 2016. See how you confirm or change your appointment below.
If you do not wish to participate, please cancel the appointment on the website or give us a call.

Before the health examination
Before the health check, we ask you to answer some questions about your health habits (takes about 10 minutes). The questions can be found at www.tjekdithelbred.dk (only available in Danish).

Randers Health Centre staff can assist you in filling in the questionnaire.

You can read more in the leaflet enclosed or at www.tdh.randerssundhedscenter.dk (only available in Danish).

Yours sincerely

General practitioner, Jens Jensen
Nordre gade 3
8900 Randers C

How do I confirm or make a new appointment?

Go to www.tjekdithelbred.dk
Key: 1234
Password: 567

Confirm the appointment, send the text message
heath
to 1245

You are welcome to call us at +45 89 15 12 15 on weekdays from 7.30 to 16.30 (Friday until 15.00).

Check your Health

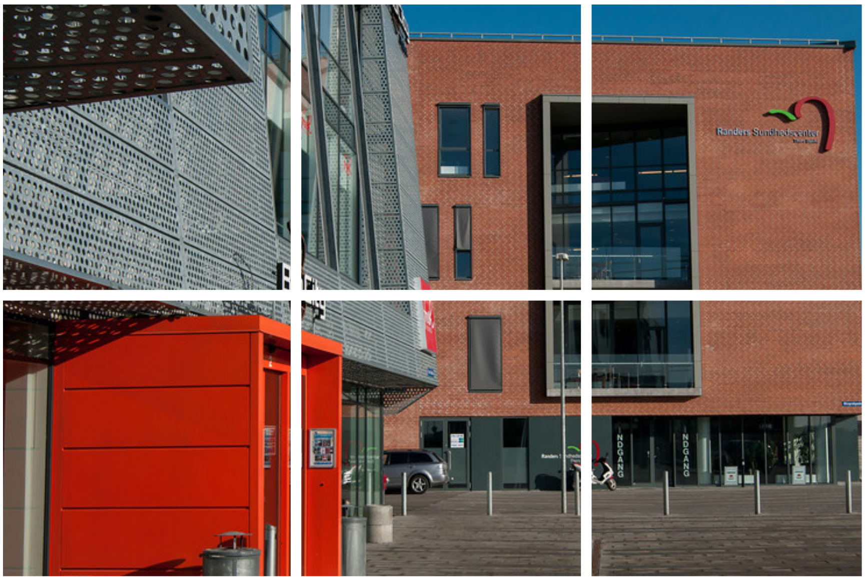

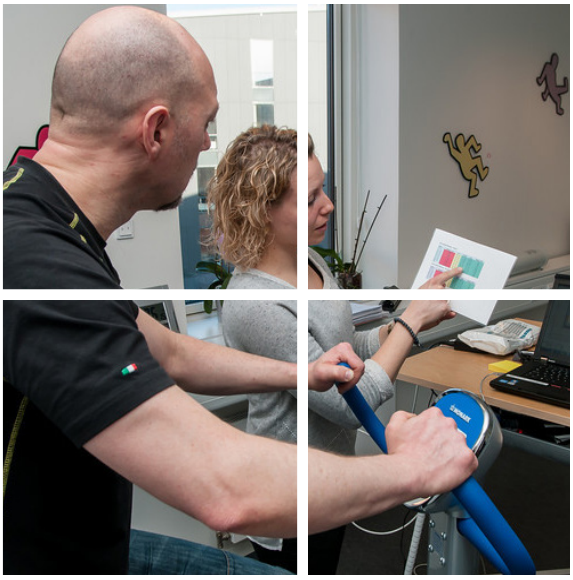

This year we
have extra focus
on examining
your lungs

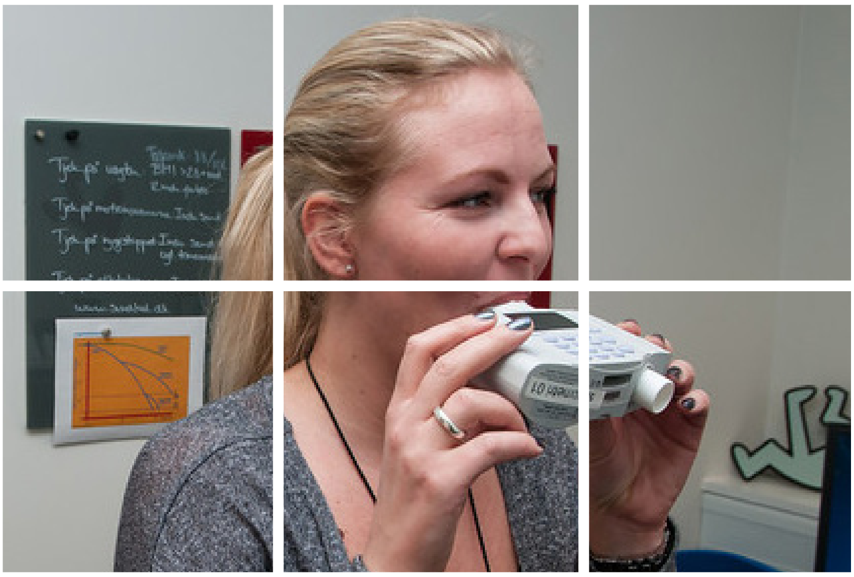

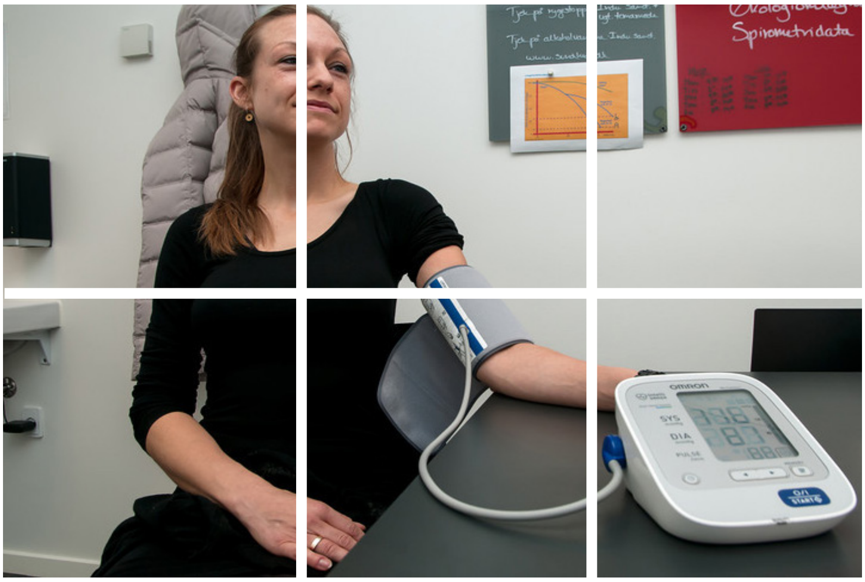

Tjek dit helbred
Randers Sundhedscenter

Check your Health and have your lung function tested

If you are born between 1962 and 1987 and live in Randers Municipality you have the possibility to get a health check. This year we have extra focus on testing your lungs and we therefore offer you a lung function test. You will be examined at Randers Health Centre and talk to your general practitioner if necessary.

Before the health examination

Before the health examination at Randers Health Centre we ask you to fill in a short questionnaire about your health habits (see invitation letter).

What happens at the health examination?

When you come for your examination at Randers Health Centre you will meet experienced staff, who will help you with the different measurements; the entire examination takes about 30 to 45 minutes.
Please wear your ordinary clothes for the examination

What is measured?

- Your height, weight and waist circumference
- Your fitness level (on an exercise bike)
- Your lung function (breathing test)
- Your blood pressure
- Your cholesterol level and blood sugar level (by a drop of blood taken from your fingertip)

After the examination you can bring your test results with you home.

What is a lung function test?

By making a simple lung function test you can find most of the early signs of lung disease (asthma and COPD). During the test you breathe into a mouthpiece and your lung function is measured. The measurement reveals if your lung function is normal or reduced.

What happens after the health examination?

If your test results show that they may be some health issues, your general practitioner would like to have a consultation with you. You need to make the appointment with your doctor yourself.
The doctor will have the results from the examination and your response to the questionnaire and you will go over both together.
If it is relevant you can participate in a number of offers at Randers Health Centre on e.g. weight loss, exercise habits, alcohol habits and smoking. After the health check you can talk to the staff at Randers Health Centre about this.

Lessons learnt from the health checks are important

First of all this is an offer to you to have a health check. Randers Municipality and Aarhus University collaborate on studying if this initiative improves the general health status of the population.
We only use the results of citizens who have consented to this. This project and the management and storage of data comply with the health laws and are approved by the Danish Data Agency (2012-41-0183).

Practical information

Randers Health Centre Thors Bakke
Biografgade 3
8900 Randers C

Tel: +45 89 15 12 15
e-mail: sundhedscenter@randers.dk
www.randerssundhedscenter.dk

Parking
Free parking for two hours at the top floor of the parking facilities at Randers Health Centre; you can enter the parking facilities from Møllegade and Thorsgade.

Buses
Buses number 3 and 4 stop at Markedsgade approx. 300 meters from Randers Health Centre.

Bicycle parking
You can park your bicycle in the parking facilities at the Randers Health Centre.

midt regionmidtjylland

De praktiserende læger
Randers og omegn

Randers Kommune

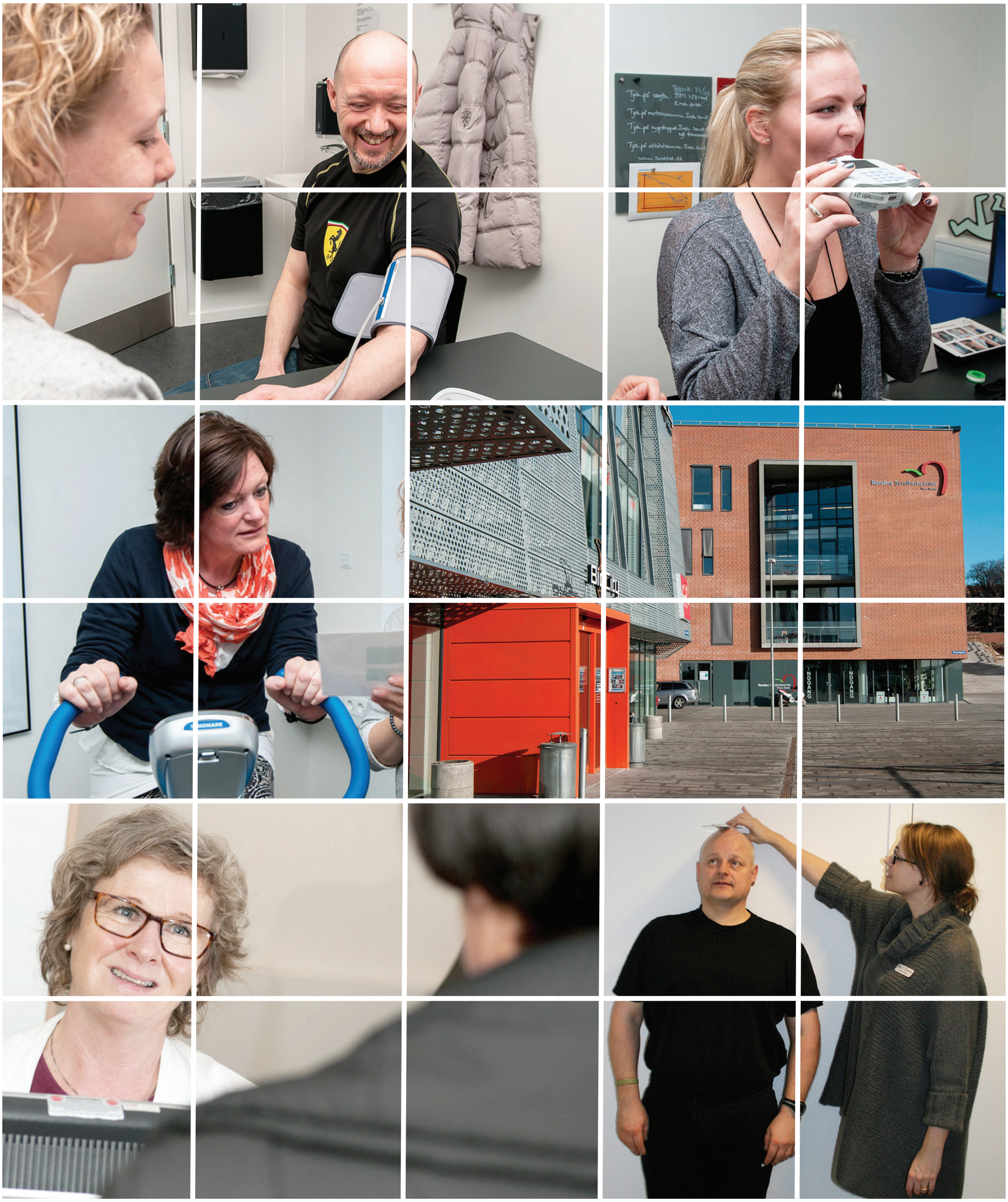

Visit Randers Health Centre and have a health check

Tjek dit helbred
Randers Sundhedscenter

Check your Health

If you are born between 1962 and 1987 and live in Randers Municipality you have the possibility to get a health check. You will be examined at Randers Health Centre and talk to your general practitioner if necessary.

Before the health examination

Before the health examination at Randers Health Centre we ask you to fill in a short questionnaire about your health habits (see invitation letter).

What happens at the health examination?

When you come for your examination at Randers Health Centre you will meet experienced staff, who will help you with the different measurements; the entire examination takes about 30 to 45 minutes.

Please wear your ordinary clothes for the examination

What is measured?

- Your height, weight and waist circumference
- Your fitness level (on an exercise bike)
- Your lung function (breathing test)
- Your blood pressure
- Your cholesterol level and blood sugar level (by a drop of blood taken from your fingertip)

After the examination you can bring your test results with you home.

What happens after the health examination?

If your test results show that they may be some health issues, your general practitioner would like to have a consultation with you. You need to make the appointment with your doctor yourself.

The doctor will have the results from the examination and your response to the questionnaire and you will go over both together.

If it is relevant you can participate in a number of offers at Randers Health Centre on e.g. weight loss, exercise habits, alcohol habits and smoking. After the health check you can talk to the staff at Randers Health Centre about this.

Lessons learnt from the health checks are important

First of all this is an offer to you to have a health check. Randers Municipality and Aarhus University collaborate on studying if this initiative improves the general health status of the population.

We only use the results of citizens who have consented to this.

This project and the management and storage of data comply with the health laws and are approved by the Danish Data Agency (2012-41-0183).

Practical information

Randers Health Centre Thors Bakke
Biografgade 3
8900 Randers C

Tel: +45 89 15 12 15
e-mail: sundhedscenter@randers.dk
www.randerssundhedscenter.dk

Parking
Free parking for two hours at the top floor of the parking facilities at Randers Health Centre; you can enter the parking facilities from Møllegade and Thorsgade.

Buses
Buses number 3 and 4 stop at Markedsgade approx. 300 meters from Randers Health Centre.

Bicycle parking
You can park your bicycle in the parking facilities at the Randers Health Centre.

midt
regionmidtjylland

De praktiserende læger
Randers og omegn

Randers Kommune

Lung function test

From 19 October 2015 to 19 December 2016, we have specific focus on testing and examining your lungs with a lung function test in Check your Health.

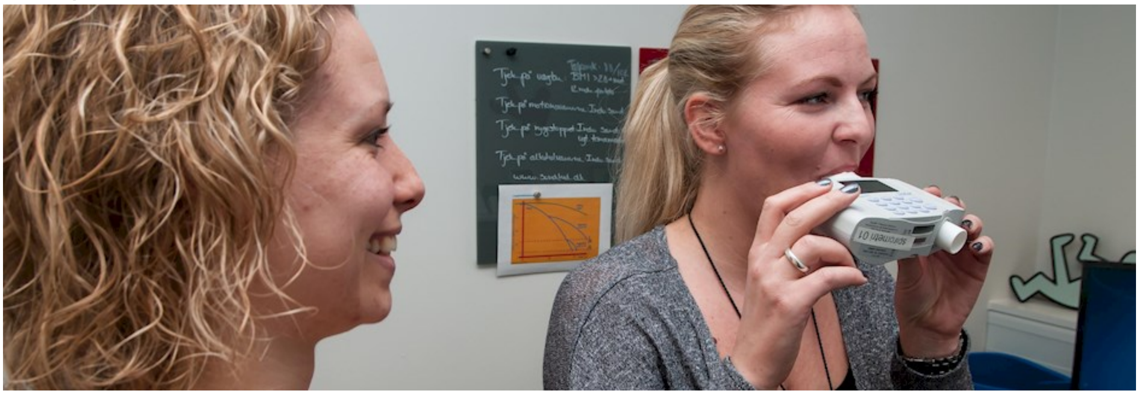

A simple measurement of how your lungs work can find most of the early signs of lung diseases (asthma and COPD). During the test you breathe into a mouthpiece and your lung function is measured. The measurement reveals if you lung function is normal or reduced.

How to take care of your lungs

Every day you breathe around 20,000 times and you can contribute yourself to keep your lungs healthy and prevent lung diseases:

- Stop smoking and avoid exposure to tobacco smoke. That is the best you can do for your lungs
- Ventilate the air at home and at your workplace
- Breathe in fresh air every day
- Be physically active every day
- Eat a healthy and varied diet

If you need help to quit smoking, please contact Randers Health Centre.

Link (only available in Danish)

Source: Lungeforeningen (Danish Lung Association)
